# Phosphorylation of Transcription Factor Specificity Protein 4 Is Increased in Peripheral Blood Mononuclear Cells of First-Episode Psychosis

**DOI:** 10.1371/journal.pone.0125115

**Published:** 2015-04-27

**Authors:** Raquel Pinacho, Gregory Saia, Montserrat Fusté, Iria Meléndez-Pérez, Victoria Villalta-Gil, Josep Maria Haro, Grace Gill, Belén Ramos

**Affiliations:** 1 Unitat de recerca, Fundació Sant Joan de Déu, Parc Sanitari Sant Joan de Déu, Universitat de Barcelona, Centro de Investigación Biomédica en Red de Salud Mental, CIBERSAM, Sant Boi de Llobregat, Barcelona, Spain; 2 Department of Developmental, Molecular, and Chemical Biology, Tufts University School of Medicine, Boston, Massachusetts, United States of America; 3 Cell, Molecular and Developmental Biology Program, Sackler School of Biomedical Sciences, Tufts University School of Medicine, Boston, Massachusetts, United States of America; IIBB/CSIC/IDIBAPS, SPAIN

## Abstract

**Background:**

Altered expression of transcription factor specificity protein 4 (SP4) has been found in the postmortem brain of patients with psychiatric disorders including schizophrenia and bipolar disorder. Reduced levels of SP4 protein have recently been reported in peripheral blood mononuclear cells in first-episode psychosis. Also, SP4 levels are modulated by lithium treatment in cultured neurons. Phosphorylation of SP4 at S770 is increased in the cerebellum of bipolar disorder subjects and upon inhibition of NMDA receptor signaling in cultured neurons. The aim of this study was to investigate whether SP4 S770 phosphorylation is increased in lymphocytes of first-episode psychosis patients and the effect of lithium treatment on this phosphorylation.

**Methods:**

A cross-sectional study of S770 phosphorylation relative to total SP4 immunoreactivity using specific antibodies in peripheral blood mononuclear cells in first-episode psychosis patients (n = 14, treated with lithium or not) and matched healthy controls (n = 14) by immunoblot was designed. We also determined the effects of the prescribed drugs lithium, olanzapine or valproic acid on SP4 phosphorylation in rat primary cultured cerebellar granule neurons.

**Results:**

We found that SP4 S770 phosphorylation was significantly increased in lymphocytes in first-episode psychosis compared to controls and decreased in patients treated with lithium compared to patients who did not receive lithium. Moreover, incubation with lithium but not olanzapine or valproic acid reduced SP4 phosphorylation in rat cultured cerebellar granule neurons.

**Conclusions:**

The findings presented here indicate that SP4 S770 phosphorylation is increased in lymphocytes in first-episode psychosis which may be reduced by lithium treatment in patients. Moreover, our study shows lithium treatment prevents this phosphorylation in vitro in neurons. This pilot study suggests that S770 SP4 phosphorylation could be a peripheral biomarker of psychosis, and may be regulated by lithium treatment in first-episode psychosis.

## Introduction

Increasing evidence indicates that transcription factor specificity protein 4 (SP4) may play a role in major psychiatric disorders. SP4 is a zinc finger transcription factor that binds to G-C/T rich sequences, regulating the expression of a large number of genes implicated in diverse biological functions [1]. SP4 is highly expressed in neurons and brain [2–4] and reduction of SP4 leads to defects in developmental dendrite patterning, hippocampal long-term potentiation, and animal behaviors associated with psychiatric disorders, including deficits in contextual and spatial memory and prepulse inhibition [5–8]. Genetic variations at the human SP4 locus have been associated with bipolar disorder and schizophrenia [9, 10]. In addition, previous studies have shown altered SP4 protein abundance in the postmortem cerebellum and prefrontal cortex both in bipolar disorder [11] and schizophrenia [12], as well as in the hippocampus in schizophrenia [13]. Thus, these studies indicate that the regulation of SP4 protein levels and function in the brain may be relevant to the pathophysiology of psychiatric disorders with psychotic features.

Reduced N-Methyl-D-Aspartate (NMDA) receptor function has been proposed as one of the main contributors to symptom emergence in schizophrenia [14–16]. NMDAR inhibition modulates SP4 protein levels in the hippocampus in a pharmacological mouse model of acute psychosis [13]. Inhibition of NMDA receptor signaling also promotes phosphorylation of SP4 protein at serine 770 (S770) in cerebellar granule neurons; this phosphorylation reduced Sp4 function independent of an effect on Sp4 levels [17]. Notably, relative levels of phosphoSp4 (pSp4) are increased in the postmortem cerebellum of bipolar disorder subjects and, in this context, SP4 S770 phosphorylation and total SP4 immunoreactivity showed an inverse correlation [18]. Moreover, we have also recently shown that relative abundance of pSP4 in the postmortem cerebellum of patients with schizophrenia directly associates with the severity of negative symptoms [18]. A reduction in SP4 immunoreactivity in peripheral lymphocytes has been reported in first-episode psychosis patients (FEP) [19]. In a more recent study, this lower peripheral SP4 immunoreactivity in FEP has been shown to correlate positively with right hippocampal volume, measured by voxel-based morphometric analysis on brain structural magnetic resonance images [20], thus suggesting a link between brain alterations found in this disorder [21] and peripheral SP4 immunoreativity. However, whether SP4 phosphorylation is increased in early psychosis, and whether this correlates with reduced Sp4 levels in lymphocytes of FEP patients is not known.

SP4 levels are increased in cerebellar granule neurons upon treatment with the mood stabilizer drug lithium [11]. As noted above, SP4 phosphorylation and protein abundance display an inverse correlation in the cerebellum of bipolar disorder patients and in cerebellar granule neurons under depolarizing or non-depolarizing conditions [17] suggesting that maybe lithium could reduce SP4 phosphorylation. Lithium is an effective treatment for bipolar disorder from the early phases of acute mania, the most efficient drug for long-term preventive treatment and it also has an anti-suicidal effect [22, 23]. Therapeutic effects of lithium only become apparent after weeks of treatment [24]; this delayed response suggests that lithium-dependent changes in gene expression programs, perhaps some mediated by SP4, contribute to long-term cellular remodeling.

Based on these previous observations, we hypothesized that SP4 S770 phosphorylation may be altered in peripheral blood mononuclear cells in first-episode psychosis patients. The main aim of this study was to investigate whether phosphorylation at SP4 S770 was increased in peripheral blood mononuclear cells in first-episode psychosis, how it related to SP4 protein abundance, and if this phosphorylation could be modulated by treatment with lithium. To test this possibility, we included a subgroup analysis based on the presence of lithium treatment in our human sample. Moreover, to further explore the effect on SP4 S770 phosphorylation of lithium and other prescribed drugs found in the FEP cohort, we tested the effect of lithium, valproic acid, and olanzapine (the most prevalent antipsychotic in our cohort of FEP) in an in vitro cell model.

## Materials and Methods

### Subjects and clinical assessments

Fourteen first-episode psychosis patients from the acute psychiatric ward of Parc Sanitari Sant Joan de Deu and fourteen similar age-sex-healthy controls were recruited and evaluated in a previously described study [19]. Inclusion criteria for patients were the presence of positive psychotic symptoms such as hallucinations or delusions for no longer than 6 months, severe enough to warrant admission to the Acute Inpatient Ward. The age at admission was between 17 and 40 years. Patients were recruited one week before discharge and evaluated by a trained psychiatrist with Positive and Negative Syndrome Scale (PANSS), Young Mania Rating Scale (YMRS) and Calgary Depression Scale for Schizophrenia (CDSS) as detailed previously [19]. A group of healthy controls of comparable age and gender distribution was recruited from the same sociodemographic environment. Exclusion criteria for controls included history of mental disorder or prescription of any psychoactive medication, and history of severe mental disorder in a first-degree relative. Additional exclusion criteria for both groups were: brain injury, neurological disease and substance abuse or dependence. All subjects gave written informed consent after a full explanation of the study. The study was approved by the Institutional Review Board of Parc Sanitari Sant Joan de Déu and the Institutional Ethics Committee of Fundació Sant Joan de Déu. Clinical assessments and blood sample collection were performed within two weeks after discharge. All patients, with the exception of one, were undergoing medication with second-generation antipsychotic drugs at the time of the blood draw. The distribution of antipsychotic treatment was as follows: 57.14% (n = 8) olanzapine, 21.43% (n = 3) risperidone, 14.29% (n = 2) aripiprazole, 14.29% (n = 2) quetiapine. 64.29% (n = 9) of the patients were also treated with mood stabilizers: 46.86% (n = 6) lithium or 21.43% (n = 3) valproic acid.

### Peripheral blood mononuclear cell samples

Peripheral blood mononuclear cells (PBMC) were obtained as described [19]. 30 ml blood was collected in EDTA tubes. PBMC were immediately isolated by Ficoll-Paque gradient method (GE Healthcare Bio-Sciences AB, Uppsala, Sweden). Briefly, 6 ml of blood sample were added to 6 ml of Ficoll-Paque reagent and centrifuged at 2000 g for 30 minutes without brake. The PBMC intermediate cell layer was collected and washed with RPMI medium (Invitrogen, Paisley, UK). The isolated cells were immediately frozen in liquid nitrogen for protein lysates. Samples were immediately stored at -80°C.

### Cell culture and treatments

Cerebellar granule neurons were obtained from P6 rats (Charles River Laboratories Inc., Wilmington, MA, USA) and cultured in 25mM KCl as previously described [25]. All protocols regarding the use of animals were approved by the Committee for the Humane Use of Animals at Tufts University School of Medicine. At DIV 4–5, cells were treated for two hours with serum-free media containing 25mM KCl and the following: lithium chloride (Sigma, St Louis, MO, USA), sodium valproate (Sigma), or olanzapine (Sigma), and protein was analyzed by Western blot.

### Immunoblotting

Protein lysates from human PBMC were extracted as described [19]. 30 μg from the same human protein lysates analyzed in our previous report [19] were analyzed in a blinded manner. Cerebellar granule neurons were lysed in RIPA buffer supplemented with protease inhibitors (Roche Molecular Biochemicals, Indianapolis, IN, USA) and phosphatase inhibitors (Santa Cruz Technology, Santa Cruz, CA, USA). Equal amount of total protein extracts were resolved by SDS⁄PAGE electrophoresis and immunoblotted with the indicated antibodies: pSP4 (specifically recognizes S770 phosphorylated Sp4) [17], SP4 (Santa Cruz Technology), pTauS396 (Cell Signaling Technologies, Beverly, MA, USA), mitogen-activated protein kinase (MAPK) p42/44 (Cell Signaling Technologies), phospho glycogen synthase kinase 3 beta (GSK3β) S9 (Cell Signaling Technologies), glyceraldehyde 3-phosphate dehydrogenase (GAPDH) (Millipore Corporation, Bedford, MA, USA) and β-actin (Sigma). Linear range exposures were quantified by densitometry using Quantity One software (BioRad) in duplicate samples for human studies or Fiji software for cerebellar granule neuron experiments.

For human samples, four independent western blots were prepared with the same amounts of the same protein extracts for each human subject; immunostaining for pSP4 and total SP4 were detected in two blots each and normalized to β-actin values from the same membrane. We calculated pSP4/SP4 ratio by referring the mean values of pSP4 immunoreactivity from two independent immunoblot analyses to the mean values of total SP4 immunoreactivity from other two independent immunoblot analyses. Both pSP4 and total SP4, normalized to β-actin, were referred to a standard sample to allow comparison of relative levels between different sets of samples and between repetitions. Total levels of SP4 were reported in [19]. The coefficient of variation of β-actin immunoreactivities for pSP4 analysis, SP4 analysis and between both analysis were calculated and validate this method of normalization (pSP4 immunoblot analysis: mean = 0.07, percentile 75^th^ = 0.08, minimum and maximum [0.01, 0.21]; SP4 immunoblot analysis: mean = 0.06; percentile 75^th^ = 0.12; minimum and maximum [0.01, 0.28]; between both analysis: mean = 0.06; Percentile 75^th^ = 0.10; minimum and maximum [0.01, 0.21]). For cerebellar granule neuron experiments, pSP4 and SP4 values from the same protein extracts were determined; SP4 values were normalized to GAPDH and phospho-SP4/total SP4 ratio was calculated and normalized to untreated, control, conditions.

### Statistical Analysis

For the analysis of PBMC samples from first-episode psychosis and control subjects D’Agostino & Pearson omnibus normality test was carried out to test whether the variables followed a normal distribution. Outliers were detected where indicated using Pierce’s criterion (as simplified by Gould) [26]. Non parametric Mann-Whitney test for quantitative variables or Chi-Square test (χ^2^-test) for categorical variables were used to compare FEP and control groups, and FEP treatment subgroups. Bivariate analyses were carried out to detect association of our variables with potential confounding factors (age, gender, college education, duration of the illness and daily antipsychotic dose), using Mann–Whitney test for two groups, and Spearman or Pearson correlation for non parametric and parametric quantitative variables. For studies of treatment effect on pSP4/SP4 in cerebellar granule neuron cultures, all results represent the mean ± SEM from at least 3 independent experiments. ANOVA followed by Bonferroni’s post hoc test for multiple comparisons were performed where indicated. Statistical analysis was performed with GraphPad Prism version 5.00 and SPSS 17. All statistical tests were two-tailed except where otherwise indicated and significance level was set to 0.05.

## Results

### Demographic and clinical characteristics

The demographic and clinical characteristics for healthy subjects and patients with first-episode psychosis were previously reported [19] and are summarised in Table 1. The total sample of patients did not show any significant differences from the healthy control group in sociodemographic variables, with the exception of college education, that was more frequent in control group (Table 1) [19]. Clinical scores for CDS and YMRS scales showed significant differences between patients and controls (Table 1). Further, a subgroup analysis of FEP group according to the presence of lithium treatment did not show any significant differences in sociodemographic or clinical variables between subgroups (Table 1).

**Table 1:** Demographic and clinical features of cases (n = 28).

	Controls (n = 14)	FEP patients (n = 14)	Statistics	pvalue
Age (years)	25.6 ± 5.2	25.2 ± 5.5	91.00^a^	0.765
Gender (male)	50.00% (n = 7)	50.00% (n = 7)	0.00; 1^b^	1.000
College education	78.6% (n = 11)	35.7% (n = 5)	5.25; 1^b^	0.022
Duration of illness (days)	N/A	81.3 ± 51.3	N/A	N/A
Daily APd^c^ (mg/day)	N/A	358.3 ± 170.3	N/A	N/A
Treatment	N/A		N/A	N/A
Antipsychotics		92.86% (n = 13)		
Antipsychotics + Li		42.86% (n = 6)		
Antipsychotics + VA		21.43%(n = 3)		
PANSS total score	N/A	77.0 ± 10.4	N/A	N/A
CDS total score	0.5 ± 1.4	5.4 ± 5.0	31.00^a^	0.001
YMRS total score	11.5 ± 1.2	14.6 ± 2.4	21.00^a^	0.000
	Lithium untreated FEP(n = 8)	Lithium treated FEP (n = 6)	Statistics	pvalue
Age (years)	23.9 ± 3.4	27.0 ± 7.5	19.00^a^	0.573
Gender (male)	62.50% (n = 5)	33.33% (n = 2)	1.17; 1^b^	0.280
College education	37.5% (n = 3)	33.33% (n = 2)	0.26; 1^b^	0.872
Duration of illness (days)	73.38 ± 59.50	91.83 ± 40.77	17.00^a^	0.401
Daily APd^c^ (mg/day)	300.0 ± 173.7	436.1 ± 143.1	12.50^a^	0.152
PANSS total score	78.5 ± 12.8	75.0 ± 6.3	22.50^a^	0.897
CDS total score	5.09 ± 6.2	4.7 ± 3.4	22.50^a^	0.897
YMRS total score	15.0 ± 2.7	14.0 ± 1.9	19.00^a^	0.559

Adapted from [19]. Mean ± standard deviation or relative frequency are shown for each variable; FEP, first-episode psychosis; APd, antipsychotic dose; PANSS, Positive and Negative Syndrome Scale; CDS, Calgary Depression Scale; YMRS, Young Mania Rating Scale; Li, lithium; VA, valproic acid; N/A, not applicable.

^a^ Mann-Whitney U is shown for non-parametric variables.

^b^ χ^2^ and degrees of freedom are shown for qualitative variables.

^c^ Chlorpromazine equivalent dose was calculated based on the electronic records of drug prescriptions of the patients [29].

### Phospho-SP4 S770 immunoreactivity is increased in peripheral blood mononuclear cells of patients with first-episode psychosis

**Fig 1:**
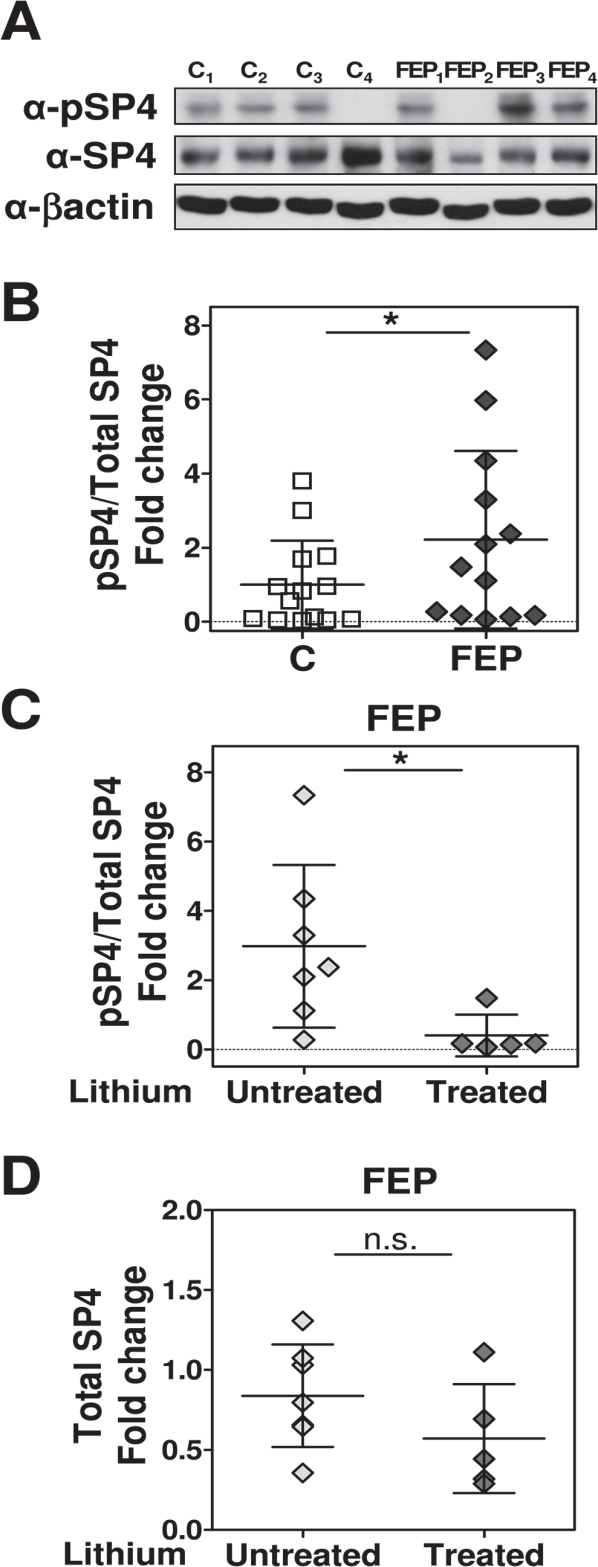
Phospho-SP4 S770 is increased in peripheral blood mononuclear cells of patients with first-episode psychosis compared to control subjects and this increase is not present in those patients with lithium prescription. Immunoreactivity of phospho-SP4 S770, SP4 and β-actin was determined in the same protein extracts from peripheral blood mononuclear cells (PBMC) of control (C, n = 14), and first-episode psychosis (FEP, n = 14) individuals. The resultant bands were quantified by densitometry. Both phospho-SP4 (pSP4) and SP4 were normalized to β-actin values and referred to a standard sample in two independent immunoblot analysis for pSP4 and SP4. pSP4/SP4 ratio was calculated for these samples by referring the mean normalized phospho-SP4 immunoreactivity levels to the mean normalized total SP4 immunoreactivity. Final pSP4/SP4 values were normalized to the mean of control group. Levels of total SP4 immunoreactivity normalized to β-actin were reported previously [19]. **(A)** Images show representative pSP4, SP4, and β-actin immunoblots from four control individuals and four FEP subjects. Patients FEP_2_ and FEP_4_ were both receiving lithium treatment. **(B)** The graph shows the mean and standard deviation of the normalized pSP4/SP4 ratio for control and FEP groups. Each value represents the mean of two independent analyses. One sample in FEP group had to be excluded from the analysis due to almost undetectable levels of pSP4 and SP4 (n = 13). Statistical analysis was performed using one-tailed Mann-Whitney test. **(C)** The graph shows the mean and standard deviation of normalized pSP4/SP4 ratio for FEP patients without lithium treatment (Untreated, n = 7), and FEP patients with lithium treatment (Treated, n = 6). Each value represents the mean of two independent analyses. An outlier was detected for pSP4/SP4 ratio in the lithium treated group using the Peirce criterion and therefore excluded from the analysis (n = 5). Statistical analysis was performed using two-tailed Mann-Whitney test. **(D)** The graph shows the mean and standard deviation of normalized total SP4 immunoreactivity for FEP patients without lithium treatment, and FEP patients with lithium treatment. Each value represents the mean of two independent analyses. Statistical analysis was performed using two-tailed Mann-Whitney test. (*p<0.05; n.s., not significant).

We determined SP4 S770 phosphorylation levels in protein lysates from PBMC of 14 FEP subjects and 14 matched healthy controls (Table 1). We calculated pSP4/SP4 ratio in these samples by referring SP4 phosphorylation levels to total SP4 immunoreactivity as determined in the same protein extracts reported in our previous study [19]. Both pSP4 and total SP4 were independently normalized to actin and a reference sample as indicated in the methods section. One sample had to be excluded from the analysis due to low detection for both SP4 and pSP4 in the FEP group (n = 13), however the statistical power for the remaining samples was 0.91 for a two-tailed test which is over the standard threshold of 0.80. We observed that the ratio of pSP4/SP4 was significantly increased in FEP subjects [U = 56.00; p = 0.047; median (IQR): control = 0.70 (0.07–1.72), FEP = 1.48 (0.17–3.82)] (Fig 1A and 1B and S1 Fig). The greater dispersion observed in FEP group suggested that other factors may be influencing pSP4/SP4 in these patients. We searched for a possible correlation of the ratio of phosphorylated SP4 to the total abundance of SP4 protein. pSP4/SP4 did not significantly correlate with total SP4 immunoreactivity (Spearman’s r = -0.230; 95% confidence interval = -0.596, 0.176; p = 0.250).

We further searched for an effect of lithium treatment on SP4 phosphorylation in these patients. We divided the FEP group into two treatment subgroups according to the presence or absence of lithium treatment. We found significantly reduced levels of pSP4/SP4 ratio in the lithium treated FEP group compared to the lithium untreated FEP group [U = 2.00; p = 0.0101; median (IQR): lithium untreated FEP group = 2.38(1.11–4.35); lithium treated FEP group = 0.17 (0.10–0.83)], (Fig 1C). In contrast, analysis of SP4 immunoreactivity revealed no significant differences when segregating FEP subjects by lithium treatment [U = 10.00, p = 0.268; median (IQR): lithium treated FEP group = 0.44 (0.30–0.90); and FEP without lithium treatment = 0.80 (0.65–1.07)] (Fig 1D).

We also investigated whether pSP4/SP4 ratio associated with the degree of severity measured by PANSS, CDS and YMRS scales in FEP patients. We found that pSP4/SP4 ratio did not correlate with the scores of any scale (Total PANSS: Spearman r = -0.199; 95% confidence interval = -0.670, 0.386; p = 0.496. Total CDS: Pearson r = -0.047; 95% confidence interval = -0.564, 0.496; p = 0.872. Total YMRS: Pearson r = -0.099; 95% confidence interval = -0.599, 0.455; p = 0.736), suggesting that the observed increase in pSP4/SP4 is not influenced by symptom severity.

We analyzed the influence of other demographic variables such as age and gender on the ratio of pSP4/SP4 in the control-FEP comparison, and in an intragroup analysis of FEP subjects designed to control for possible effects on pSP4/SP4 ratio of duration of the illness and daily antipsychotic dose. This analysis showed that pSP4/SP4 ratio is not significantly associated with any of the potential confounding factors (Table 2).

**Table 2:** Influence of demographic and clinical characteristics on pSP4/SP4 ratio in peripheral blood mononuclear cells in first-episode psychosis and control subjects.

	pSP4/SP4 ratio	
	FEP-C cohort	FEP cohort
Age		
Spearman r	-0.091	-0.483
95% CI	(-0.465, 0.310)	(-0.833, 0.146)
p value	0.652	0.112
Gender		
Male (Mean ± SD)	1.68 ± 2.09	2.74 ± 2.55
Female (Mean ± SD)	1.48 ± 1.83	0.74 ± 0.86
U	89.00	8.00
p value	0.942	0.149
College education		
No (Mean ± SD)	1.90 ± 2.24	1.90 ± 2.46
Yes (Mean ± SD)	1.34 ± 1.69	2.95 ± 2.42
U	69.00	13.00
p value	0.317	0.504
Duration of illness^a^	N/A	
Spearman r		0.095
95% CI		(-0.521, 0.646)
p value		0.770
Daily APd^a^ ^,^ ^b^	N/A	
Spearman r		-0.269
95% CI		(-0.739, 0.378)
p value		0.399

FEP, first-episode psychosis; C, control; CI. confidence interval; SD, standard deviation; U, Mann-Whitney U; APd, antipsychotic dose; N/A, not applicable.

^a^ Analysis in first-episode psychosis group only.

^b^ Daily Chlorpromazine equivalent dose.

### Lithium reduces the levels of phospho-SP4 S770 in rat cultured cerebellar granule neurons

**Fig 2:**
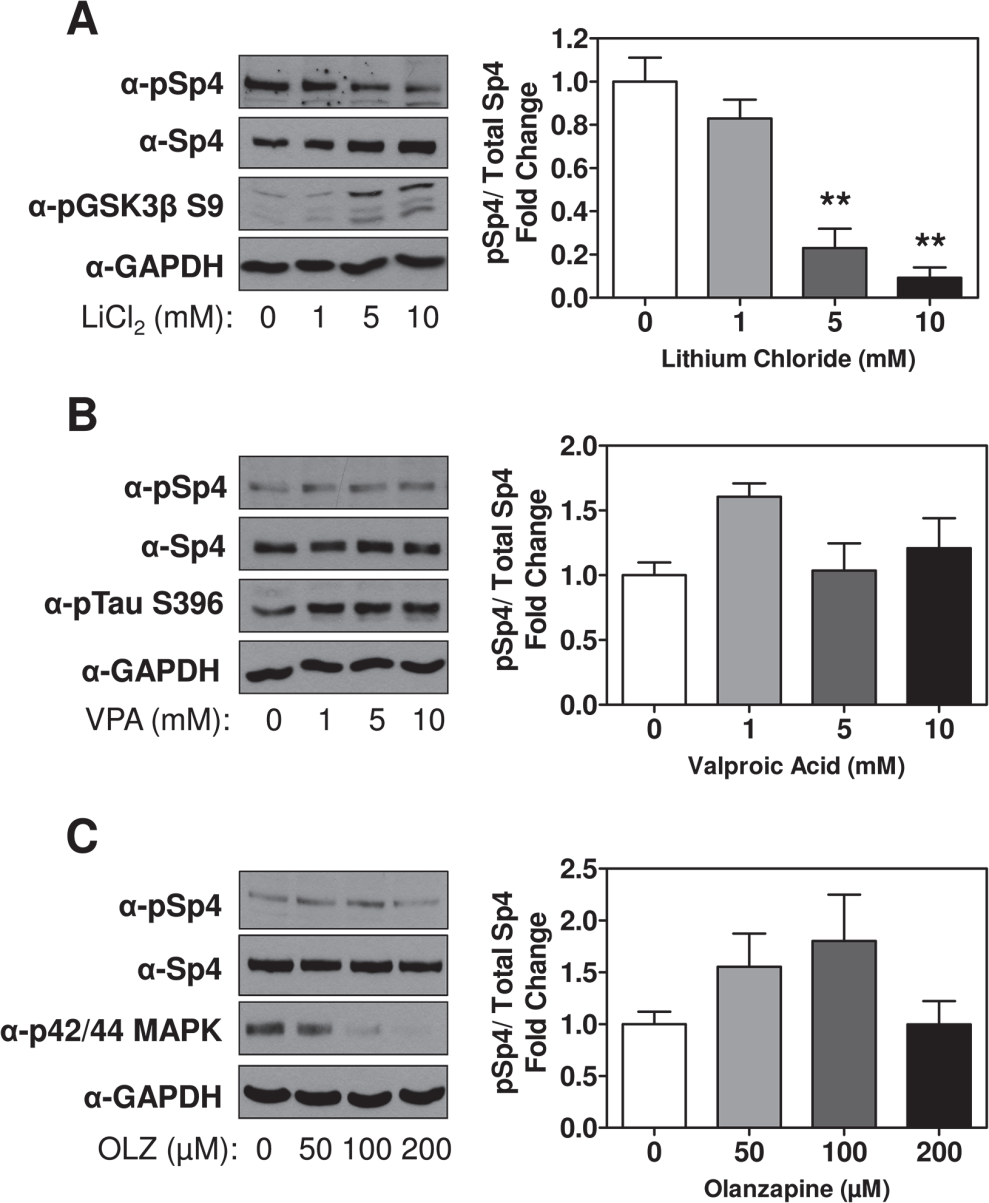
SP4 phosphorylation at S770 is reduced by lithium chloride treatment. **A.** Cerebellar granule neurons were treated with serum-free media containing 25mM KCl for two hours in the presence of the indicated concentrations of lithium chloride **(A)**, valproic acid (VPA) **(B)** or olanzapine (OLZ) **(C)**. 14 μg **(A)** or 5 μg **(B and C)** of a total protein extracts were analyzed by Western blot using antisera to phospho-SP4 S770, total SP4, phospho-GSK3β S9 (a positive control for lithium treatment) **(A)**, phospho-Tau S396 (a positive control for VPA) **(B)**, p42/44 MAPK (a positive control for OLZ) **(C)** and GAPDH as a loading control. The graphs show the densitometric quantification of S770 phosphorylated SP4 relative to the levels of total SP4. Statistical analysis was performed using ANOVA followed by Bonferroni’s post hoc comparison between untreated condition and the different doses of drugs. N = 3 (**p<0.01).

To further explore the effect of lithium and other drugs prescribed in the FEP cohort on SP4 phosphorylation, we used rat primary cultured cerebellar granule neurons as an in vitro cell model. We tested if lithium chloride, valproic acid or olanzapine, the most frequently prescribed antipsychotic in our cohort, influenced the levels of SP4 S770 phosphorylation in cultured cerebellar granule neurons. We found that only treatment with lithium chloride significantly decreased pSP4/SP4 ratio (ANOVA: F = 20.4; df = 3, 8; p = 0.0004) (Fig 2A and S2 Fig); while pSP4/SP4 ratio was not influenced by valproic acid (ANOVA: F = 2.60; df = 3, 12; p = 0.101) or olanzapine (ANOVA: F = 1.771; df = 3, 8; p = 0.230) in cultured neurons (Fig 2B and 2C and S2 Fig).

## Discussion

Our study shows that phosphorylation of transcription factor SP4 at S770 is increased in peripheral blood mononuclear cells of first-episode psychosis patients, that lithium-treated patients have reduced SP4 phosphorylation levels and that lithium suppresses SP4 phosphorylation in an in vitro cell model. Thus, our findings suggest that SP4 phosphorylation is a potential biomarker for first-episode psychosis which may be regulated by lithium treatment.

Our data adds further evidence for a role for SP4 phosphorylation at S770 in the post-translational regulation of SP4 in the context of psychiatric disorders. An increasing body of evidence indicates that SP4 transcription factor has a role in complex psychotic disorders through its regulation of a large network of genes, including components of NMDAR signaling pathways [8, 11–13, 19, 27, 28]. In line with these studies, we have described alterations of SP4 in the postmortem brain of psychotic patients which supports a role for SP4 in these disorders. In particular, we have shown reduced immunoreactivity of SP4 in the postmortem cerebellum and prefrontal cortex of subjects with bipolar disorder [11]; a negative association of SP4 immunoreactivity with negative symptom severity in the cerebellum of patients with schizophrenia [12]; and altered SP4 levels in the postmortem hippocampus of subjects with schizophrenia and in a mouse model of acute psychosis [13]. We also recently reported that lower SP4 immunoreactivity in first-episode psychosis patients correlates positively with right hippocampal volume, measured by voxel-based morphometric analysis on brain structural magnetic resonance images [20]. Most relevant to the work described here, we have observed that SP4 phosphorylation at S770 inversely associated with SP4 immunoreactivity in human postmortem cerebellum [18] and this phosphorylation is increased in this region in bipolar disorder patients and in schizophrenia patients with more severe negative symptoms, which both showed reduced SP4 immunoreactivity [11, 12]. Here we report that SP4 S770 phosphorylation is increased in peripheral lymphocytes of first-episode psychosis. In this context, however, we observed that SP4 phosphorylation did not correlate with total SP4 immunoreactivity. This is similar to the effect of NMDAR inhibition in cultured neurons, which increased SP4 S770 phosphorylation and inhibited Sp4 function, without an effect on total SP4 immunoreactivity [17]. Thus, increased SP4 phosphorylation in lymphocytes of FEP may reflect alterations in upstream signaling pathways that control SP4 functions.

Lithium has been successfully used as a mood stabilizer for the treatment of bipolar disorder for decades, and, to date, remains the most efficient drug for long-term preventive treatment [22, 24]. In our study, we found that FEP patients that had received lithium treatment exhibited reduced SP4 S770 phosphorylation in lymphocytes when compared to FEP patients that had not received lithium and that lithium treatment in vitro suppressed SP4 phosphorylation in cultured rat cerebellar granule neurons. This preliminary finding that lithium seems to inhibit SP4 phosphorylation in patients may suggest that this phosphorylation could be a readout measure of the treatment. However, further studies are needed to propose this phosphorylation as a marker for treatment effectiveness and a possible therapeutic target in psychotic patients. In addition, since these patients were treated with other drugs, it remains possible that the observed reduction in pSP4 in this subset of patients could be due to other treatments. However, our analysis in rat cerebellar granule neurons supports the idea that lithium may be responsible for the reduction of pSP4 in patients, and not olanzapine, the most prevalent prescribed antipsychotic in this cohort. Future studies are needed to fully explore the effect of other treatments on this phosphorylation in blood cells.

In peripheral lymphocytes from patients, total SP4 immunoreactivity was not significantly affected by lithium treatment, suggesting that the pathways regulating SP4 phosphorylation and protein abundance are independent in human lymphocytes. In contrast, we previously reported that, in cerebellar granule neurons, lithium partially stabilized SP4 protein, leading to significantly increased protein levels in non-depolarizing conditions [11]. Thus our data suggests that, in cerebellar granule neurons, lithium impacts pathways that regulate both SP4 stability and phosphorylation at S770, consistent with the view that these pathways are coupled in some contexts. Identifying these pathways is a future goal for better understanding the signaling pathways regulating SP4 and their possible contribution to the pathophysiology and therapy of psychiatric disorders.

Analysis of biological parameters in first-episode psychosis is helpful to identify altered players that could be involved in the etiopathogenesis of psychotic disorders in the early stages. We have recently described an increase in SP4 phosphorylation in the postmortem cerebellum of bipolar disorder and schizophrenia patients [18]. The finding that the pSP4/SP4 ratio is increased in FEP and can also be observed in the cerebellum in a much later stage of the disease, supports the possibility that this modification in PBMC could be a readout of SP4 alteration occurring in the brain in the early stages of the disease. However, we cannot rule out the possibility that SP4 S770 phosphorylation may be dynamic and could change over the course of the disease in PBMC, as we also cannot predict how peripheral levels of phosphorylated SP4 relate to the actual levels in the different brain areas involved in psychosis.

A few limitations need to be acknowledged. The sample size analysed here is small and the findings require replication in a larger sample of healthy subjects and FEP patients treated and untreated with lithium. In addition, our first-episode psychosis cohort is not drug naïve since the patients had been receiving antipsychotic treatment. Nevertheless, treatment duration was shorter than 6 months in all cases, and our statistical analysis did not show a correlation of SP4 phosphorylation with the daily dose of antipsychotics suggesting that these treatments may not influence our results. Moreover, our study of treatment effect on SP4 phosphorylation in cultured rat cerebellar neurons showed that SP4 phosphorylation levels were not affected by in vitro treatment with the antipsychotic olanzapine, the most representative antipsychotic in our cohort (n = 8). Despite these limitations, findings from this study may contribute towards a better understanding of the molecular mechanisms that underlie psychosis. Peripheral blood screening of SP4 phosphorylation levels may be a useful peripheral biomarker of psychosis and potentially regulated by lithium treatment in the early stages of psychosis.

## Supporting Information

S1 Fig Phospho-SP4 S770 is increased in peripheral blood mononuclear cells of patients with first-episode psychosis compared to control subjects. Immunoreactivity of phospho-SP4 S770 (pSP4), SP4 and β-actin was determined in the same protein extracts from peripheral blood mononuclear cells (PBMC) of control (C, n = 14), and first-episode psychosis (FEP, n = 14) individuals as previously reported [19]. Images show full size representative immunoblots from phospho-SP4 S770 **(A)**, total SP4 **(B)** and β-actin **(A and B)** immunoreactivity. N/A, not applicable.
(TIF)

S2 Fig SP4 phosphorylation at S770 is reduced by lithium chloride treatment. Cerebellar granule neurons were treated with serum-free media containing 25mM KCl for two hours in the presence of the indicated concentrations of lithium chloride **(A)**, valproic acid (VPA) **(B)** or olanzapine (OLZ) **(C)**. Images show full size immunoblots from phospho-SP4 S770, total SP4, phospho-GSK3β S9 (a positive control for lithium treatment), phospho-Tau S396 (a positive control for VPA) **(B)**, p42/44 MAPK (a positive control for OLZ) **(C)** and GAPDH immunoreactivity.
(TIF)

S1 Table Detailed demographic, clinical features and molecular measures of cases. FEP, first-episode psychosis; APd, antipsychotic dose; PANSS, Positive and Negative Syndrome Scale; CDS, Calgary Depression Scale; YMRS, Young Mania Rating Scale; ARP, aripripazole; QTP, quetiapine; Li, lithium; OLZ, olanzapine; RS, risperidone; VA, valproic acid; N/A, not applicable.
(XLSX)
